# Unveiling the Hurdles in Cultivating Humanistic Physicians in the Clinical Setting: An Exploratory Study

**DOI:** 10.21315/mjms2020.27.3.12

**Published:** 2020-06-30

**Authors:** Rita Mustika, Diantha Soemantri

**Affiliations:** 1 Department of Medical Education, Faculty of Medicine, Universitas Indonesia, Jakarta, Indonesia; 2 Doctoral Program in Medical Sciences, Faculty of Medicine, Universitas Indonesia, Jakarta, Indonesia

**Keywords:** humanistic physician, medical humanism, professionalism, role model

## Abstract

**Background:**

The importance of cultivating a humanistic physician has gained attention in medical education. Humanistic values are established in early education and medical schools should provide a suitable environment to nurture and grow these values into professional identity. The clinical setting has a significant impact due to its direct involvement of students in real-life situations.

**Objectives:**

The present study aims to explore the hurdles in cultivating humanistic physicians in the clinical setting.

**Methods:**

We conducted a qualitative study involving medical students in the clinical phase, as well as residents, clinical teachers, and module administrators in the clinical setting under study.

**Results:**

Respondents from different groups of stakeholders shared the same definition for ‘humanistic physician’: a physician who provides patient-centred care while demonstrating empathy, respect, compassion, integrity, knowledge, competence and a collaborative spirit. Despite changes in the healthcare system and technological advancements, humanistic physicians are still needed.

**Conclusion:**

Cultivating humanistic physicians is a complex process, requiring various methods and assessments. Role models play a significant role in this process, which included not only clinical teachers but also peers. Feedback from peers was perceived as an important factor. The key hurdles identified were negative role models, and a less humanistic learning environment and the students’ personal backgrounds.

## Introduction

The profession of physician is one that requires both ‘the head and the heart.’ The head refers to the physician’s biomedical and clinical knowledge and skills, while the heart represents the art of caring (1). During medical practice, physicians apply their knowledge and skills to solve patients’ health problems. The relationship between physicians and patients is based on trust, as patients come to physicians because they believe that physicians have the necessary knowledge and expertise to cure their diseases. Patients also expect physicians to display humanistic behaviours; that is, patients seek physicians who are kind, show compassion and treat them well (2).

There has recently been concern that physicians are becoming less humanistic (3), for which the use of artificial intelligence and other advanced technologies in medicine is considered to be a primary reason. Advanced technological devices have changed the relationship between physicians and patients in the clinical setting. For example, using an electronic medical record during the physician–patient encounter busies the physician with typing tasks, thus decreasing eye contact between physician and patient. Some advanced technological devices require patient interaction without the physician’s help, further limiting the connection between physician and patient. Information about diseases is also widely available online (4). Furthermore, some changes in the healthcare system have caused physicians to feel obliged to see more patients in their clinics, which has sometimes prevented physicians from providing proper humanistic care. In addition to needing to meet with more patients, paperwork has increased, leading to patient complaints (5).

Humanism is considered ‘a way of being’ that is demonstrated by the internal values through which an individual interacts with the world and those around them. These values tend to evolve into the attributes of integrity, honesty, care and compassion. Professionalism, in contrast, is ‘a way of acting’ that manifests in how physicians treat their patients, including the abilities to communicate effectively, employ biomedical and clinical competencies and take a humanistic approach towards patients. Humanism is a core feature of medical professionalism; without humanistic qualities, physicians tend to practise fake professionalism and unprofessional behaviour can then occur more easily when there is pressure in the physician’s personal life (6).

A study by Papadakis et al. (7) shows that unprofessional behaviours by physicians are highly related to unprofessional behaviours during the educational period. Spurred by concerns about increasing unprofessional behaviours, the medical education community has begun exploring how to teach humanism and professionalism. The clinical setting offers a precious opportunity to develop humanistic values, as students are directly involved in the community of healthcare providers in a real medical environment. In the present study, we seek to explore the process of teaching humanism and professionalism in the clinical setting from the perspectives of clinical teachers, residents and medical students.

## Methods

This qualitative study follows a phenomenological design. Data was obtained from focus group discussions (FGD) and in-depth interviews conducted by the authors. The respondents were chosen via maximum variation sampling from four groups in the Academic Health System at Universitas Indonesia (AHS-UI): clinical-phase medical students, residents, junior clinical teachers and senior clinical teachers. To gather a comprehensive dataset, respondents with a variety of ages, genders and clinical specialisations were chosen from each group. We conducted eight FGDs—two within each group—and two in-depth interviews with only the clinical teachers who had organised the humanism-related modules in the clinical setting being studied.

Data was collected and transcribed verbatim, then analysed via thematic analysis using the Steps for Coding and Theorisation method (8). Data was coded according to its source, with ST for clinical-phase students, R for residents, CT1–CT2 for senior clinical teachers and CT3–CT4 for junior clinical teachers.

**Figure 1:**
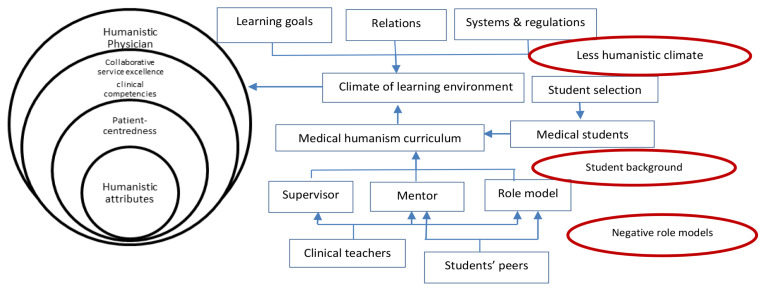
The process of cultivating humanistic physicians in the clinical setting

The AHS-UI, where this study was conducted, is a part of the faculty of medicine of UI and houses nine academic hospitals that form the academic health system. The programme under study is an undergraduate medical education programme with a 3.5-year academic phase and a 2-year clinical phase. Following the current standards of medical education, this programme implements a competency-based curriculum. There are seven areas of competency that must be achieved by the time students graduate, which include professionalism, self-awareness and personal development, effective communication, medical knowledge, information technology, clinical skills and disease management. The competencies related to humanism (i.e. professionalism, effective communication and self-awareness and personal development) were taught during the study period as separate modules in the academic phase and integrated modules in the clinical phase. Efforts to cultivate humanistic physicians by the medical school included a student selection process that screened for humanistic attributes and a grand lecture by the senior professor of medicine held at the beginning of the programme, which served as a welcome to students entering the professional community of medical physicians. During the study period, methods for cultivating humanistic physicians varied from the provision of lectures and small group discussions to role play, field visits and reflective practice (Figure 1).

In each semester of the academic phase, there was a two-credit module dedicated to teaching empathy, ethics and professionalism, with each module having a different learning objective related to professional development and the assessment being mostly reflective writing or observation reports. In the clinical phase, teaching professionalism was integrated into other content teaching; although learning objectives were defined, there were no special sessions dedicated to teaching professionalism. The clinical teacher was expected to discuss content related to professionalism while discussing clinical cases, and assessments related to professionalism were integrated into other workplace-based assessments.

## Results

Themes identified via data analysis included descriptions and the need for humanistic physicians in today’s society; how to cultivate humanistic physicians in the clinical setting; role modelling and mentoring; and the hurdles in cultivating humanistic physicians in the clinical setting. The following sections discuss each theme in greater detail.

i) Descriptions and the need for humanistic physicians ‘
  … they treat patients as humans, not just a disease. They give solutions for all the patient’s problems, not just their medical problems…’ (CT2)
  ‘…they put patient needs over material needs, and even their own needs…’ (R1)

Groups of respondents described the humanistic physician as one who treats their patients holistically and comprehensively; they treat not only the disease, but also the patient as a whole. The medical students emphasised that the humanistic physician is one who puts patients’ needs over their own. In contrast, the clinical teachers emphasised the ability to collaborate as a critical attribute. The clinical teachers also emphasised the need to know the healthcare system and the financial ability to solve the problems that their patients face. The senior clinical teacher group added that a humanistic physician should be able to anticipate and understand patients’ underlying needs even before the patients express them. Therefore, the attributes of a humanistic physician proposed by the respondents included patient-centeredness, altruism, a collaborative spirit, knowledgeability, competence, attentiveness, caring and compassion.

‘… Humanistic physicians are needed more than before because patients can access information [about their diseases] more easily now, so they need doctors who can answer their concerns and relieve the anxiety caused by information that they learned about through the internet…’ (CT4)

‘…Yes, a humanistic physician is still needed because life is harder [and] there are a lot of poor people in society; they need doctors who put their needs over material [needs]…’ (R6)

‘…Society still needs humanistic physicians, because human touch cannot be replaced by technological developments…’ (CT4)

The groups of respondents agreed that humanistic physicians are still needed—perhaps even more so in the modern era. The advancement of information technology has allowed more convenient access to information online, which can lead to information overload. Patients need physicians who can provide them with proper explanations and help them to understand the information they find, thus making that information beneficial to their healthcare. The respondents in the medical student group highlighted poverty as a significant barrier to proper care; low-income patients need physicians who can help them regardless of their financial ability. Another factor is that human touch cannot be replaced by technology.

ii) Strategies to cultivate humanistic physicians in the clinical setting
  ‘…Peer support in [conducting] humanistic behaviours towards patients…’ (R5)
  ‘…Small group discussions on professionalism, reflective practice…’ (R3)

FGDs among the medical student and resident groups revealed that their peers were the most influential factor in developing their humanistic characteristics. Having peers that reminded them to treat patients as human beings helped them to become humanistic physicians, as the desired behaviours would eventually become habits. The group of residents highlighted the importance of more structured learning in small group discussions and reflective practice for learning professionalism.

‘…With stadium general [grand lecture]: first [provide a] lecture from a senior professor in medicine to welcome and introduce students to [the] physician community…’ (CT2)

‘…Role modelling and mentoring [is essential] … students see the teacher and imitate their humanistic qualities…’ (CT4)

Clinical teachers agreed that the grand lecture from a senior professor in medicine at the beginning of the study period was still the most important teaching method. The senior professor who gave the lecture would give a mental model of a competent physician that the students would become in the future, while junior clinical teachers discussed other roles of a clinical teacher besides that of role model, such as a mentor and supervisor.

All groups agreed that teaching medical students to become humanistic physicians is a process that requires a nurturing approach, starting with the student selection process and followed by providing a suitable atmosphere that is conducive to humanistic values and behaviours. The clinical teachers highlighted that implementing assessments that measure specific humanistic values and behaviours profoundly influences the cultivation process. The in-depth interviews with the module teams uncovered the evaluation of various efforts to cultivate humanistic physicians; the teams also mentioned the need for faculty development in preparing clinical teachers to teach professionalism.

iii) Role modelling and mentoring
  ‘…Clinical teachers play a significant role as role models. Students will see and act like them…’ (CT1)
  ‘…Clinical teachers need to explain humanistic behaviour and ask students to act accordingly…’ (CT3)
  ‘…Clinical teachers should give feedback to students, so they know what is right and what is wrong…’ (R1)

According to both the junior and senior groups of clinical teachers, the clinical teacher plays a significant role in cultivating humanistic behaviour. They argued that role models are essential in providing positive examples that students can imitate. The clinical teacher not only models how a physician should treat their patients, but also shows how to treat others, including students. In addition to their service as role models, the clinical teachers also mentioned their roles as mentors and supervisors and noted that clinical teachers must identify and understand any generational gaps that might exist between themselves and their students, then adjust their approach accordingly. During the in-depth interviews, the module teams mentioned that faculty development initiatives could equip the clinical teachers with the tools necessary to perform their roles as role models, mentors and supervisors.

‘…Our friends who always remind us to treat patients as human beings…[to] care and show compassion… they help develop [humanistic qualities] in us…’ (ST4)

The medical student and resident groups also agreed that positive role modelling by clinical teachers is essential and further highlighted the importance of having peers as role models and mentors. Senior students who show a positive attitude towards humanistic behaviours can encourage junior students to behave the same way. Having peers and seniors who offer reminders to take a humanistic approach to patient care also has a positive influence on cultivating humanistic physicians.

iv) The hurdles in cultivating humanistic physicians
  ‘…Not every doctor shows humanistic behaviours; sometimes, we saw doctors mistreating patients… not listening to them… communicating [in only] one direction…’ (R3)
  ‘…Some doctors put their managerial tasks over their patients’ [needs]. They left their patients [waiting] in [the] clinic [to participate in a] management meeting…’ (R1)

All groups mentioned that negative role models—both clinical teachers and peers—are the most prominent hurdles in cultivating humanistic physicians. Role models who mistreat patients, fail to practise empathy and display selfish behaviours could send students the wrong message about how to treat patients professionally.

‘…The new [national insurance] system [has] made us serve more patients each day. We cannot even spend 10 minutes [caring] for one patient… how can we serve patients empathetically…’ (CT2)

The clinical learning environment itself could also pose a barrier. According to the clinical resident group, uncaring relationships in the clinical setting influence the learning climate, eventually leading to less humanistic behaviours among residents and students. The clinical teacher group highlighted the influence of the healthcare system as well, which forces physicians to treat many patients in a limited timeframe, thus increasing the risk of a less humanistic service provision.

‘…we are busy typing [medical records] facing our computer … difficult to maintain eye contact with the patient. How [can] we show empathy…’ (R2)

‘… [the learning] depends on the student’s background too… they entered [medical school] as teenagers, their personalities have developed. Parenting style and previous education must have an impact [on their characters] …’ (CT4)

Technological advancements and students’ backgrounds could also disrupt the process of cultivating humanistic physicians. The clinical teacher group explained that students enter medical school with diverse educational, economic and socio-cultural backgrounds that influence their humanistic qualities.

## Discussion

In line with the more traditional definitions, in this study, the humanistic physician is defined as one who treats the patient as a human being, not just a disease (9). Some of the attributes of humanistic physicians revealed in this study are similar to those found by Chou et al. (4), including integrity, altruism, compassion and honesty. The faculty development programme and longitudinal professionalism modules in the academic phase, which provide clinical teachers and medical students a theoretical framework to view humanistic physicians, could explain this resemblance.

Furthermore, the clinical teacher group in this study revealed that humanistic physicians are expected to solve patients’ problems holistically, not just from a medical standpoint. The humanistic physician should also be sensitive to and understand their patients’ verbal and nonverbal communication (9). Hence, a humanistic physician should also develop other skill sets, such as the willingness and ability to collaborate effectively as well as knowledge about the healthcare system and relevant data. These abilities represent the prerequisite skill set for physicians in the twenty-first century (10). All of these descriptions support a patient-centred approach to medical care, which is the current trend of medical care.

This study revealed that, although technology is developing rapidly, the need for humanistic physicians has not diminished. Due to the explosion of internet-based information, the relationship between physician and patient has changed dramatically, as physicians are no longer the sole source of medical information, and some medical interventions can even be performed by robots (11). Nevertheless, the human connection between physician and patient cannot be replaced (12). Physicians of the future must be competent in meeting patients’ needs by being fully present in the provision of care, working together with the patient to fight disease.

The teaching of medical humanism represents a form of character-building, which requires a constant flow of practice and opportunities for self-reflection (13). This study reported various teaching methods: grand lecture, small group discussions, formal reflective practice, role modelling, mentoring and peer-assisted learning. Interestingly, the clinical teachers rated highly the importance of a grand lecture by a senior professor, while the students emphasised the importance of small group learning, peer feedback and mentoring for cultivating humanistic values. This difference is probably due to generational differences between clinical teachers and students. Desy et al. (14) reported that the largest proportion of medical students are from the millennial generation, who were born between 1982 and 2000, and clinical teachers are mostly from earlier generations. Studies show that millennials differ from earlier generations, especially in their need for feedback and interaction; they also prefer mentoring and personalised learning. Less interactivity or opportunities for personalised feedback could be the reason why the grand lecture was not a method favoured by students. Nevertheless, various teaching methods, including lectures, are still needed to address humanism across settings (15).

Role modelling humanistic behaviours is a highly influential component in teaching humanism (4). All respondents agreed that clinical teachers play a significant role in developing the professional identities of medical students, especially in the clinical environment. The clinical teacher group mentioned their role as role models, mentors and supervisors in teaching humanism, but the respondent from the clinical teacher group remarked that not every clinical teacher is aware that they are being watched, as students not only learn via direct explanation but also by observing the daily actions and behaviours of their teachers. Moreover, the workload and complexity of clinical situations could inhibit a clinical teacher from becoming a positive role model. Students reported that they sometimes observed negative behaviours shown by their senior and clinical teachers while interacting with patients. Examples of behaviours that the students perceived to be negative were one-way communication with a patient, conducting examinations or interventions with a patient without informed consent, ignoring patients’ questions, failing to maintain eye contact with a patient and neglecting patients to attend managerial meetings. The students said that they tended to unconsciously follow the same behaviours and actions as their clinical teachers. Such role models send the wrong message: that these behaviours are acceptable in the medical world.

All respondents declared that, to achieve the ultimate goal of cultivating humanistic physicians, the existence of negative role models is a hurdle. As explained by Bandura’s social learning theory, students will observe the work of their clinical teachers, both passively and actively. During the passive observation, students will unconsciously incorporate any behaviours they see into their new behavioural scheme, whereas during active observation, students will actively explore the effects and values of the behaviours they see through reflection and abstraction, then translate these insights into principles, which will eventually become their new behaviours (16). Therefore, through active observation, negative role models will not always create negative behaviours in students.

The results of this study also demonstrate the power of peer-assisted learning. According to students and residents, role modelling is not only provided by clinical teachers, but also by peers. Peers who display humanistic behaviours serve as an excellent example to the whole group. However, according to the negativity bias theory, negative behaviours are more natural to learn (16). If a peer shows less humanistic behaviours, other students are at higher risk of adopting them.

On the other hand, mentoring and supervision are considered to be significant roles of clinical teachers in the clinical setting. These are slightly different from role modelling; in mentoring and supervision processes, teachers actively teach their students, give feedback to them and monitor their development (17). To perform these roles effectively, clinical teachers must have a skill set that includes giving constructive feedback, the ability to practice self-reflection and the ability to teach these skills, as students and residents both agreed that mentoring is pivotal to their humanistic learning. By giving feedback to each other, peers also play the role of mentor to a certain extent. The students in this study believed that feedback from peers was a significant help in cultivating humanistic behaviours.

The respondents also reported other hurdles in cultivating humanistic physicians, including a less humanistic climate in the learning environment and the personal backgrounds of the students. The learning environment could become a hurdle if the goals of learning, the clinical facilities and the relationships between people and systems in the environment do not support humanistic behaviours (18). Examination rooms that are small, unventilated and filled with a never-ending flow of patients make students feel uncomfortable and could lead to burnout. The national health system, as it currently operates, has increased patient loads in most teaching hospitals, and interactions between physicians and patients have become shorter, which is not conducive to teaching and modelling humanistic conversation and behaviour. The use of electronic medical records has also become a burden, as this practice tends to limit physician–patient eye contact.

Relationships between people in the learning environment also influence the teaching of humanism. If students are poorly treated and experience an inhumane climate, it is likely to be more difficult for them to develop humanistic behaviours. Students will also observe the relationships between clinical teachers, patients, other health professionals and their peers; these relationships, along with the system and goals of learning, make a significant contribution to students’ perceptions of the learning environment (19).

The students themselves could also pose a hurdle in humanistic teaching. A medical student enters medical school in their late teenage years. Their parents and guardians should have already implanted the seeds of humanism (6); medical school then grows these seeds into humanistic professional behaviours by the time of graduation. Therefore, if the students’ parents and prior knowledge have failed to implant the seeds of humanism, a humanistic environment—even with positive role models—may be insufficient to cultivate humanistic physicians (6).

Students’ responses to learning vary and are likely influenced by prior education, experience and personal qualities. Therefore, medical teachers with the ability to give constructive feedback, the creation of a humanistic climate and equipping students with the ability to reflect and learn actively from a role model could solve the hurdles in the process of cultivating humanistic physicians and ultimately shape professional physicians for the future.

## Conclusion

In the current era of patient-centredness, the need for humanistic physicians is increasing, and optimising the teaching of humanism and professionalism is, therefore, a necessity. The hurdles in cultivating humanistic physicians include a less humanistic learning climate, negative role models, students’ backgrounds and the differences between clinical teachers and students in their perceptions of effective teaching and learning approaches. To optimise the teaching of humanism, a medical institution needs to create a humanistic learning climate that encourages humanistic relationship and mentoring. Students need to be able to learn from a role model actively. Furthermore, the explicit expected learning objectives related to humanism and professionalism need to be written formally into the curriculum, followed by the implementation of various learning methods and assessments that cater to the different needs of students. In terms of faculty development, clinical teachers need to be familiarised with the characteristics of the new generation of medical students and equipped with the skill of giving constructive feedback.
